# Genome-Wide Identification of the *HSF* Genes in Sweet Potato and Functional Role of *IbHSF22* in Anthocyanin Accumulation and Salt Stress Tolerance

**DOI:** 10.3390/plants15020236

**Published:** 2026-01-12

**Authors:** Chen Chen, Qing Zhang, Ying Peng, Menglai Zhou, Tayachew Admas, Lianjun Wang, Xinsun Yang, Wenying Zhang

**Affiliations:** 1 MARA Key Laboratory of Sustainable Crop Production in the Middle Reaches of the Yangtze River/Hubei Key Laboratory of Waterlogging Disaster and Agricultural Use of Wetland/Research Center of Crop Stresses Resistance Technologies, College of Agriculture, Yangtze University, Jingzhou 434025, China; 2022730066@yangtzeu.edu.cn (C.C.);; 2 Jingzhou Institute of Technology, Jingzhou 434020, China; 3 Hubei Key Laboratory of Food Crop Germplasm and Genetic Improvement/Key Laboratory of Crop Molecular Breeding, Ministry of Agriculture and Rural Affairs, Institute of Food Crops, Hubei Academy of Agricultural Sciences, Wuhan 430064, China

**Keywords:** sweet potato, heat shock transcription factor (*HSF*), anthocyanin, salt stress, transgenic tobacco, gene family

## Abstract

Heat shock transcription factors (HSFs) play a central role in mediating plant responses to abiotic stress. Anthocyanins, one of the most important secondary metabolites in plants, contribute to both stress tolerance and the enhancement in crop nutritional quality. However, the possible role of HSFs in regulating anthocyanin biosynthesis in sweet potato (*Ipomoea batatas* L.) remains unknown. This study conducted a genome-wide analysis of the sweet potato HSF gene family to explore their functions related to anthocyanin metabolism and salinity stress. Multiple stress-inducible promoter elements were identified within *IbHSF22*, including those induced by drought, salt, heat, ABA, and light. For functional characterization of this gene, a 35S-driven overexpression construct was prepared and then transformed into *Nicotiana benthamiana*. Overexpression of *IbHSF22* led to a nearly two-fold increase in anthocyanin content, concurrently with the elevated expression of key structural genes such as *NtCHS*, *NtF3H*, *NtDFR*, and *NtANS*. Under salt stress, the transgenic plants also exhibited enhanced tolerance, which was associated with maintained antioxidant enzyme activity and concerted induction of stress-responsive genes, events that collectively resulted in decreased oxidative damage. Therefore, the present work identifies *IbHSF22* as an integrator of anthocyanin biosynthesis and salt defense mechanisms. These findings provide a conceptual basis and candidate gene strategy for dual improvement in stress resilience and nutritional quality in sweet potato breeding.

## 1. Introduction

Vegetable sweet potato is a functional vegetable with high added value [1], and the tender stems and leaves contain plenty of anthocyanins, flavonoids, and dietary fiber [2]. As an important water-soluble flavonoid, anthocyanins not only give plant tissues characteristic colors like red, purple, and blue, but also have significant antioxidant, anti-inflammatory, and anti-cancer activities and show broad application prospects in the prevention of chronic diseases and promotion of human health [3]. Vegetable sweet potato is therefore hailed by the international medical community as an “anti-cancer vegetable” and has been included in the “space food” and “longevity food” systems of the United States and Japan [3,4]. It is worth mentioning that anthocyanins also play an important role in the response to abiotic stress for plants. Through scavenging reactive oxygen species (ROS), they can reduce the oxidative damage to plants and thus enhance the adaptability of plants to poor conditions such as drought, high salinity, and extreme temperature [5,6]. However, the main variety presently cultivated in China possesses significantly lower anthocyanin content and stress resistance compared to superior varieties such as the Japanese ‘Ayamurasaki’ and excellent strains from Taiwan Province of China [4]. Because the crop is regularly exposed to salt and drought stress in the field, its yields typically fall by 30–50%, a loss that seriously hampers the industry’s long-term sustainability [7]. In-depth analysis of the coordinated regulatory mechanism between anthocyanin biosynthesis and stress resistance has become a core scientific issue in the sweet potato genetic improvement strategy with respect to enhancing its quality and tolerance to abiotic stresses [8,9].

HSFs are the central regulators mediating plant responses to environmental stresses [10]. HSFs enhance plant tolerance to different abiotic stresses, such as high temperature, drought, and salinity, through activating heat shock proteins and antioxidant-related genes [11]. Based on structural features of oligomerization domains, HR-A/B [12], plant HSFs could be divided into three major classes, including A, B, and C. While class A members (e.g., *HSFA1/2*) are primary transcriptional activators that act directly on downstream stress-responsive genes [13], most class B factors usually function as repressors to modulate the activity of class A HSFs under stress conditions. However, some studies of B-class factors have indicated that B-class factors could also increase stress tolerance against abiotic stress by promoting anthocyanins or flavonoids’ accumulation [14,15,16]. The functional roles of class C remain less defined, but emerging evidence suggests their involvement in fine-tuning stress response pathways. In parallel with this, emerging studies extend the functional scope of HSFs from canonical stress responses toward the regulation of secondary metabolism, particularly anthocyanin biosynthesis [17]. For example, *HSFA2* acts as a transactivator for the key structural genes in the anthocyanin pathway, such as *ANS* and *DFR*, besides coordinating enhanced oxidative protection with increased anthocyanin accumulation through the upregulation of ROS-scavenging enzymes, including *APX1* [11,13].

Although the *HSF* family has been systematically characterized in model species such as *Arabidopsis thaliana* and *Solanum lycopersicum* L., comprehensive genomic identification and functional analysis of HSFs in hexaploid sweet potato remain limited. To date, there has been no direct evidence linking HSFs to the regulation of anthocyanin biosynthesis in sweet potatoes, and the connection between anthocyanin accumulation and tolerance to abiotic stress remains poorly understood. To address this knowledge gap, we undertook genome-wide analysis to identify the *HSF* gene family in sweet potato. Consistent with the exception noted for some B-class factors [14,15,16], our initial transcriptomic data indicated that *IbHSF22*, belonging to the B2 subgroup, is differentially expressed (log2 fold change > 4.0) between high and low anthocyanin sweet potato cultivars, suggesting that this gene may be positioned at an important regulatory node between the stress response and anthocyanin biosynthetic pathway, and could possess a divergent or condition-dependent regulatory function beyond conventional repression. Therefore, we then characterized in detail the *IbHSF22*, including its phylogeny, genomic structure, and cis-elements in its promoter region, as a means to understand more fully its role in the plant. An overexpression construct for *IbHSF22* was created and transiently expressed in *Nicotiana benthamiana*. The resulting transgenic plants were subjected to salt stress under controlled conditions, and anthocyanin levels, along with key indicators of ROS build-up and antioxidant enzyme activities, were assessed. This study investigates the dual role of *IbHSF22* in abiotic stress tolerance and anthocyanin biosynthesis, giving theoretical insights and potential genetic resources that could be used for breeding nutritionally improved, stress-resilient vegetable sweet potato cultivars.

## 2. Results

### 2.1. Identification, Characterization, and Candidate Selection of IbHSFs

**Figure 1:**
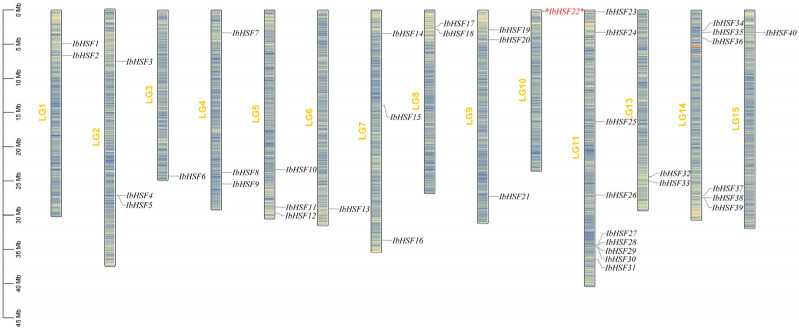
Distribution of *IbHSF* genes on sweet potato chromosomes. A total of 40 *IbHSF* genes were distributed randomly across the 15 chromosomes of sweet potato. The left side shows chromosome length in megabases (Mb); black labels represent various distributions of *IbHSF* genes, the target gene *IbHSF22* extensively studied in this work is marked with a red asterisk (*), and yellow labels denote the corresponding chromosome numbers.

Using the HMM profile of *HSF*, a total of 45 candidate *IbHSF* genes were identified from the sweet potato genome. Further confirmation of these candidate genes as *HSF* family members was performed with analyses using the NCBI conserved domain database and InterProScan, confirming 40 *IbHSF* genes. The corresponding genes were denoted as *IbHSF1* to *IbHSF40*, according to the chromosomal locations (details in Table S1). Chromosomal distribution analysis demonstrated that the 40 *IbHSF* genes showed uneven distribution within the 15 chromosomes. Most chromosomes carried only 1–3 genes, while Chr11 and Chr14 harbored 9 and 6 *IbHSF* genes, respectively. Contrarily, *IbHSF6*, *IbHSF13*, and *IbHSF22* were individually located on Chr3, Chr6, and Chr10, respectively. Interestingly, most *IbHSF* genes clustered in the vicinity of telomeres, and *IbHSF22* and *IbHSF23* demonstrated particularly close locations to chromosome ends, indicating functional significance (Figure 1). To prioritize key candidates, we leveraged prior transcriptomic evidence from our laboratory. Comparative analysis of sweet potato cultivars (Fushu No. 7–6 and EC16) with contrasting anthocyanin accumulation identified *IbHSF22* as the most differentially expressed gene within the *HSF* family. Thereby, *IbHSF22* was selected for the systematic dissection of its regulatory function in anthocyanin accumulation and salt tolerance.

Molecular weight, isoelectric point, and subcellular localization were further analyzed for the identified 40 *IbHSF* genes. The lengths of the encoded proteins ranged from 68 to 640 amino acids, while their molecular weights ranged from 7.16 kDa to 71.24 kDa. The theoretical pI values ranged from the low of 3.85 to the high of approximately 10.04, while the hydrophobicity index for IbHSF proteins was below zero, indicating that they are all hydrophilic in nature. Predictions of subcellular localization showed that IbHSF15 was localized in the cytoplasm, IbHSF4 and IbHSF27 in chloroplasts, while all other IbHSFs were mainly localized in nuclei. In conclusion, IbHSF proteins were mainly acidic, hydrophilic sulfur-containing proteins functioning mainly in the nucleus (Table S1).

### 2.2. Phylogenetic Analysis of IbHSFs

**Figure 2:**
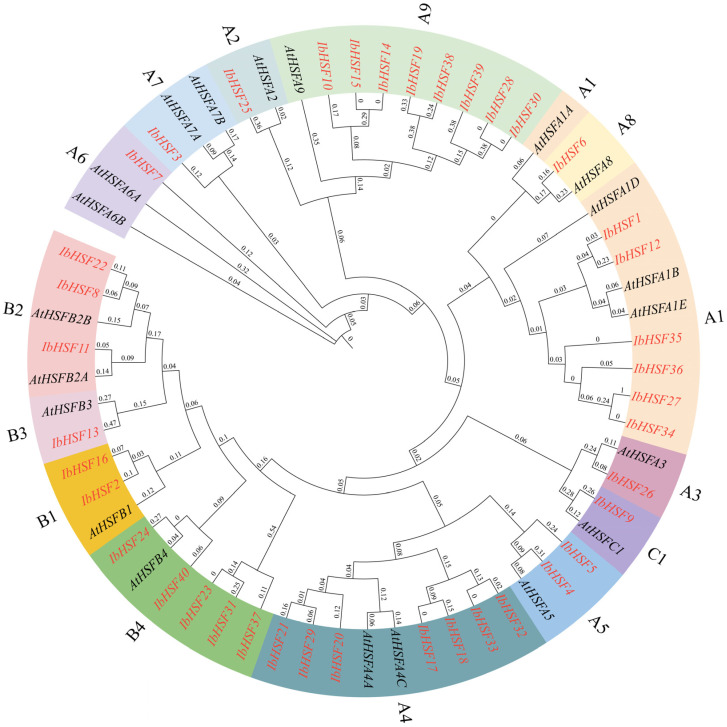
Phylogenetic tree of *HSF* genes in sweet potato and *Arabidopsis thaliana*. The phylogenetic tree was constructed by using MEGA11 software (V11.0.13) with the Maximum Likelihood method, using bootstrap values of 1000, different colors represent different *HSF* subfamilies.

To clarify the evolutionary relationships of sweet potato heat shock transcription factors (IbHSFs) and *Arabidopsis thaliana* heat shock transcription factors, a phylogenetic tree was developed for *A. thaliana* and sweet potato. This tree can be divided into three subfamilies: A, B, and C, with 21 AtHSF proteins and 40 IbHSF proteins. As indicated in Figure 2, IbHSFA represents the biggest class, including 28 members clustered in nine subgroups (A1–A9), and IbHSF22 falls into the B2 subgroup. In comparison, IbHSFB includes four subgroups (B1–B4) having a total of 11 genes, while IbHSFC is the tiny class, only containing IbHSF9.

### 2.3. Gene Structure and Conserved Domain Analysis of IbHSFs

**Figure 3:**
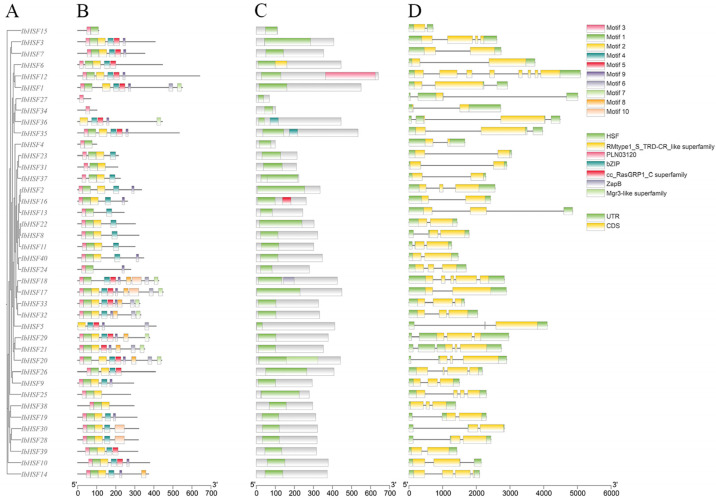
Gene structure and conserved motifs analysis of IbHSFs: (**A**) phylogenetic relationship tree; (**B**) conserved motifs of IbHSFs; (**C**) SMART conserved HSF domain; (**D**) gene structure of IbHSFs.

Gene structure and conserved motifs analysis are very important for further investigation of gene function and evolution. The gene structures of IbHSFs are quite different, as shown in Figure 3D; the number of exons varies from 1 to 8, and about 90% of IbHSFs contain 2 to 4 exons. Furthermore, there is only one exon in *IbHSF5*, *IbHSF15*, and *IbHSF27*, while *IbHSF12* has eight exons. On the aspect of UTRs, all *IbHSF* genes contain at least two UTRs. The *B2* subgroup includes three *IbHSF* members: *IbHSF8*, *IbHSF11*, and *IbHSF22*, whose gene structures are similar to each other. *IbHSF23*, *IbHSF31*, and *IbHSF37* of the B4 subgroup also show similar structures, whereas subfamily A has a highly variable structure. The sequence length is also quite different among distinct subfamilies. Gene sequences of the A1 subgroup are much longer than those of other subgroups, but the gene sequences in the B2 subfamily are quite short. This indicates that genes in the same subfamily may have similar structural features.

Analysis of the 40 IbHSF protein sequences identified ten conserved motifs with lengths ranging from 11 to 41 amino acids (Figure 3B). All IbHSF proteins, except for IbHSF5 and IbHSF36, contained Motif 3. Based on the results from the CDD database, Motif 1, Motif 2, and Motif 3 were identified as DBDs. Phylogenetic analysis showed that several motifs were specifically distributed in certain subfamilies: Motifs 5, 6, 7, 8, and 10 were specific to subfamily A. Moreover, Motif 6 was unique to subgroups A1, A4, and A5, while Motif 7 and Motif 8 co-occurred in all members of subgroup A4.

By using the SMART and Pfam databases to carry out a comprehensive search and analysis, Motif 1, Motif 2, and Motif 3 were identified as fragments of the HSF domain. The remaining motifs had no specific annotations. The presence of Motifs 1, 2, and 3 in all IbHSF proteins coincided with the formation of the very conserved HSF DBD region, which suggests that these motifs may play important roles in the biological functions of HSF proteins Figure 3C.

### 2.4. Gene Duplication and Collinearity Analyses of IbHSFs

**Figure 4:**
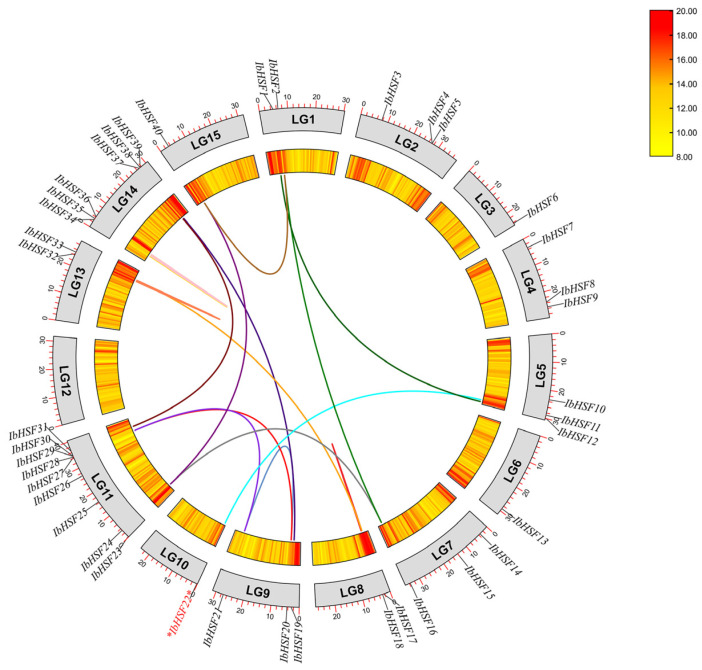
Chromosomal distribution and inter-chromosomal relationships of *IbHSF* genes; the target gene *IbHSF22* extensively studied in this work is marked with a red asterisk (*), colored lines represent duplicated *IbHSF* gene pairs.

In order to investigate the contribution of gene duplication events to the expansion of the IbHSFs, we have conducted intra-genomic synteny analysis of sweet potato chromosomes. The result is visualized in a Circos plot and presented in Figure 4. We identified 17 pairs of segmentally duplicated IbHSFs, represented by colored linking lines within this figure. These collinear gene pairs are dispersed on 10 out of the 15 chromosomes, indicating that large-scale segmental duplication is one of the main driving forces responsible for the expansion of the IbHSFs family. Remarkably, collinear gene pairs are highly dense on several chromosomes. For instance, LG9 shows extensive collinearity with multiple chromosomes, such as LG11 and LG14. Other connections were found between *IbHSF17* and *IbHSF18* on LG8; *IbHSF32* and *IbHSF33* on LG13; and *IbHSF34*, *IbHSF35*, and *IbHSF36* on LG14. These support the fact that the described chromosomes may function as hotspots for historical genomic rearrangements and duplications that together shape the current genomic structure of the IbHSFs family. These segmentally duplicated gene pairs surely provide valuable information regarding the evolutionary history of the gene family under consideration.

**Figure 5:**
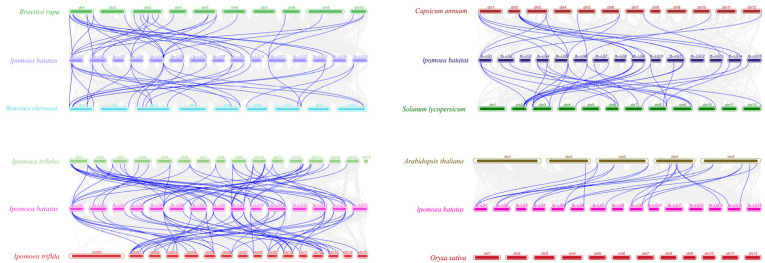
Collinearity analysis of the sweet potato genome with eight plant genomes shows that the gray lines represent collinear blocks, and the blue lines indicate collinear *HSF* gene pairs.

To study the evolutionary history of IbHSFs, we conducted a collinearity analysis between the sweet potato *IbHSF* genes and their orthologs in eight plant species: *Ipomoea trifida*, *Ipomoea triloba*, *Arabidopsis thaliana*, *Oryza sativa*, *Brassica rapa*, *Brassica oleracea*, *Solanum lycopersicum*, and *Capsicum annuum*. The result is presented in Figure 5, collinearity analysis revealed 59 and 61 orthologous gene pairs between the sweet potato and *I. trifida* and *I. purpurea* genomes, respectively. In addition, 21 collinear gene pairs were identified with *A. thaliana*, 30 with *B. rapa*, 27 with *B. oleracea*, 34 with *S. lycopersicum*, and 23 with *C. annuum*, while no orthologous relationship was obtained with *O. sativa*. The list of all orthologous gene pairs is detailed in Table S4.

Remarkably, 18 genes in *I. purpurea* and 19 genes in *I. trifida* have collinearity with at least two *IbHSF* genes in sweet potato. Examples include: *itf02g13540.t1* in *I. purpurea*, which is collinear to *IbHSF17*, *IbHSF18*, *IbHSF32*, and *IbHSF33* in sweet potato; *itb02g08970.t1* in *I. trifida*, which is collinear to *IbHSF17*, *IbHSF18*, *IbHSF20*, *IbHSF21*, *IbHSF32*, and *IbHSF33*. For instance, *IbHSF22* in sweet potato is collinear to the sequences in *I. trifida*: *itb12g25610.t2*, *itb13g04970.t3*, and *itb08g00370.t1*, as well as *I. purpurea*: *itf08g00360.t1*, *itf12g25210.t1*, and *itf13g03340.t1*.

These results suggest that the IbHSFs may have played important roles in plant evolution. Further, the observation of more than two direct orthologs of most *HSF* genes in the sweet potato genome, both in *I. purpurea* and in *I. trifida*, strongly supports the hypothesis that sweet potato has undergone multiple whole-genome duplication events.

### 2.5. Detection of Cis-Acting Elements in the Promoters of IbHSFs

**Figure 6:**
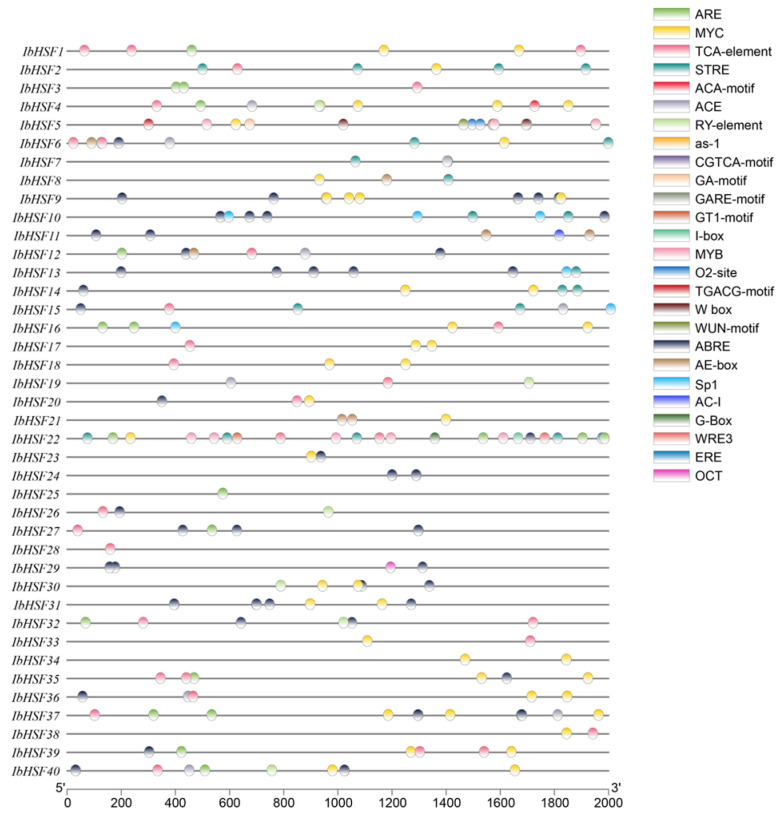
Distribution of cis-regulatory elements in the promoter regions of *IbHSF* genes. Each promoter region encompasses a 2000 bp sequence upstream of the transcription start site of the respective *IbHSF* gene. The colored boxes indicate 23 distinct types of cis-regulatory elements.

To explore the possible regulatory mechanism of *IbHSF* genes’ expression in response to abiotic stress, a systematic predictive analysis of the cis-acting elements in promoter sequences was conducted. The results are shown in Figure 6. Approximately 70% of promoter sequences have been found to contain one or more defined stress-responsive elements, mainly including anaerobic induction response elements (ARE), abscisic acid response elements (ABRE), and osmotic stress response elements (STRE). This overrepresentation of stress-related cis-elements strongly supports the functional implication of these modules in the transcriptional activation of IbHSFs during abiotic stress responses. Of all IbHSFs’ promoters, 80% contained at least one hormone-responsive element, such as ABRE and the salicylic acid-responsive TCA-element. Interestingly, *IbHSF22* contains motifs related to light response (G-Box, I-box, GT1-motif), hormone response (STRE, TCA-element, WRE3, ARE, ABRE, ERE, MYC), and stress response (MYB), suggesting that this gene integrates different signaling pathways under environmental stress. Significantly, three or more ABRE elements were found individually within the promoters of the five genes *IbHSF9*, *IbHSF10*, *IbHSF13*, *IbHSF27*, and *IbHSF31*. This high density of ABRE motifs provides strong evidence that these genes may be key regulators within the ABA-mediated signaling network, with functions considered critical for enhancing plant tolerance to abiotic stress.

### 2.6. Subcellular Localization of Selected IbHSF Proteins IbHSF22 (g38186)

**Figure 7:**
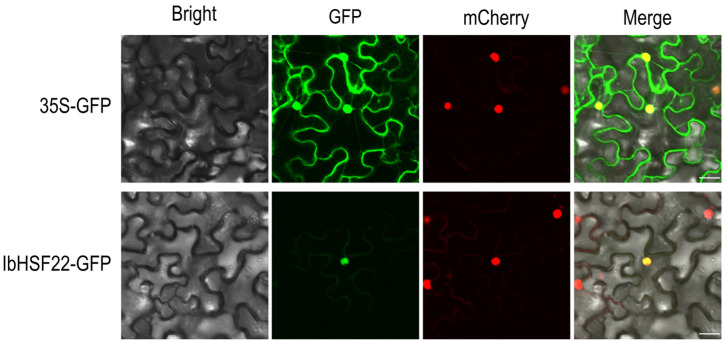
Subcellular localization detection of IbHSF22 in tobacco leaves. Scale bar = 20 μm.

To further verify the subcellular localization of the IbHSF22 protein, a 35S-GFP construct was used as a positive control and transiently expressed in tobacco leaf epidermal cells (Figure 7). Analysis through a confocal fluorescence microscope revealed that the IbHSF22-GFP fusion protein was targeted only to the nucleus, confirming the bioinformatic prediction that it acts as a transcription factor.

### 2.7. IbHSF22 Promotes Anthocyanin Synthesis in Nicotiana benthamiana

**Figure 8:**
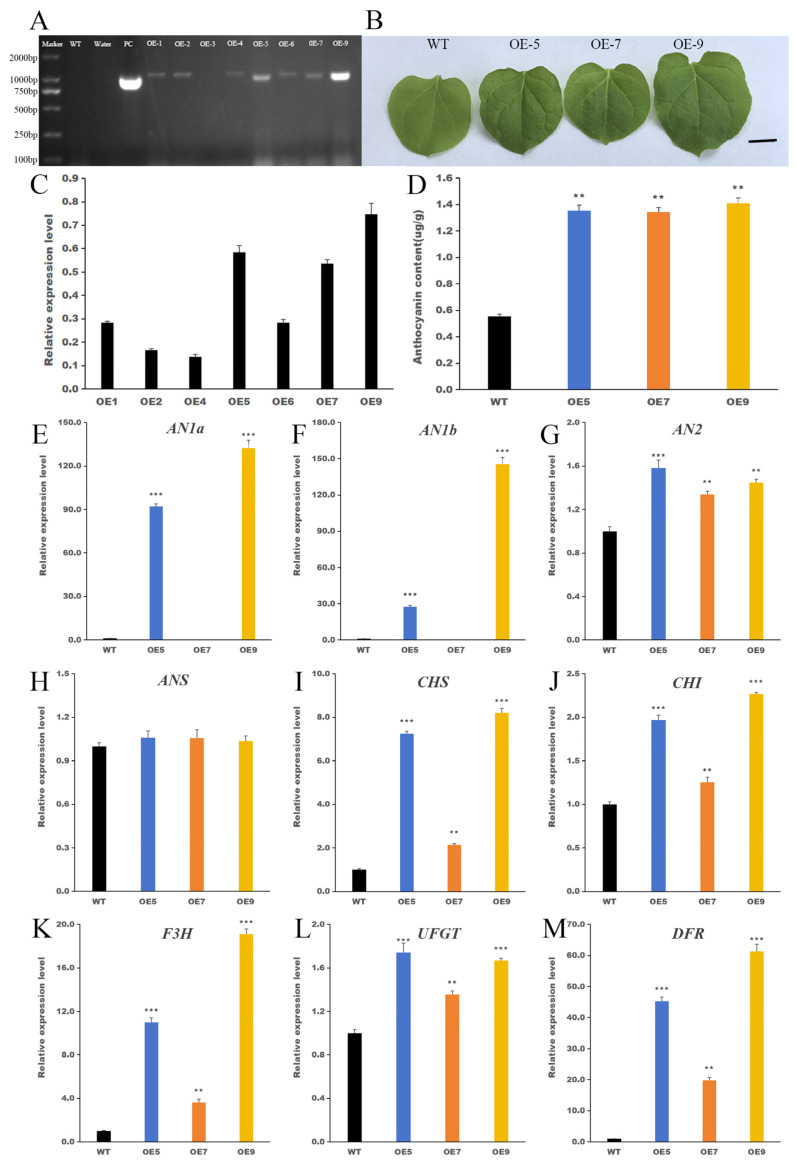
(**A**) Identification of positive transformation lines, (**B**) *IbHSF22* expression levels in seven independent lines, (**C**) Representative leaves of WT and three high-expression lines, (**D**) Anthocyanin content in WT and high-expression lines, (**E**–**M**) Expression levels of anthocyanin synthesis-related genes in control (0 d). *AN1A* and *AN1B* expression in the WT was below the qRT-PCR detection limit (Ct ≧ 40) and set to 40 for quantification. Relative expression in OE lines was normalized to the internal reference gene and calculated using the 2^−ΔΔCt^ method. ** *p* < 0.01, *** *p* < 0.001 versus the WT under identical conditions (Student’s *t*-test; mean ± SD, n = 3).

To verify the function of *IbHSF22*, stable overexpression (OE) of *IbHSF22* was achieved in *Nicotiana benthamiana*. A total of 12 independent lines were obtained through tissue culture. Eight potential positive lines were preliminarily screened out by GUS staining. Further genomic PCR detection was performed using specific primers for *IbHSF22*-ORF-R, 35S-F, and, eventually, seven independent *IbHSF22*-OE transgenic plants were identified (Figure 8A). Three relatively highly expressing lines of *IbHSF22*: *IbHSF22*-OE5, *IbHSF22*-OE7, and *IbHSF22*-OE9, were selected (Figure 8B) for additional experimental analysis. Phenotypic observations indicated that leaves of the transgenic lines were indeed significantly darker than the wild-type (WT), suggesting increased anthocyanin accumulation (Figure 8C). Given that *Nicotiana benthamiana* exhibits negligible floral pigmentation and our study focuses on leaf anthocyanin accumulation, the trait from which *IbHSF22* was originally identified, we quantified anthocyanin content in leaves. The results indicated that the anthocyanin contents in the three overexpression lines were approximately twice those of the WT with high statistical significance (*p* < 0.01) (Figure 8D). Moreover, transcriptional analysis of the anthocyanin biosynthesis pathway demonstrated that *IbHSF22*-OE acted as a potent upstream regulator (Figure 8E–M). Notably, it activated to a large event level the expression of *NtAN1a* and *NtAN1b*, which were transcriptionally silent (Ct ≧ 40) in WT plants, in transgenic lines OE5 and OE9. In parallel, the expression of key structural genes was significantly increased: the late biosynthetic gene *DFR* was markedly upregulated in all transgenic lines, with the largest induction (61.3-fold) observed in OE9, while early biosynthetic genes *CHS* and *F3H* were also strongly induced. In contrast, the transcriptional levels of *AN2*, *ANS*, *UFGT*, and *CHI* were relatively unchanged. This shows that *IbHSF22* is a potential high-level transcriptional activator that initiates the anthocyanin pathway by turning on master regulators such as *NtAN1a/b* and upregulating a few critical structural genes, thereby reprogramming anthocyanin biosynthesis.

### 2.8. Overexpression of IbHSF22 Enhances Salinity Resistance

**Figure 9:**
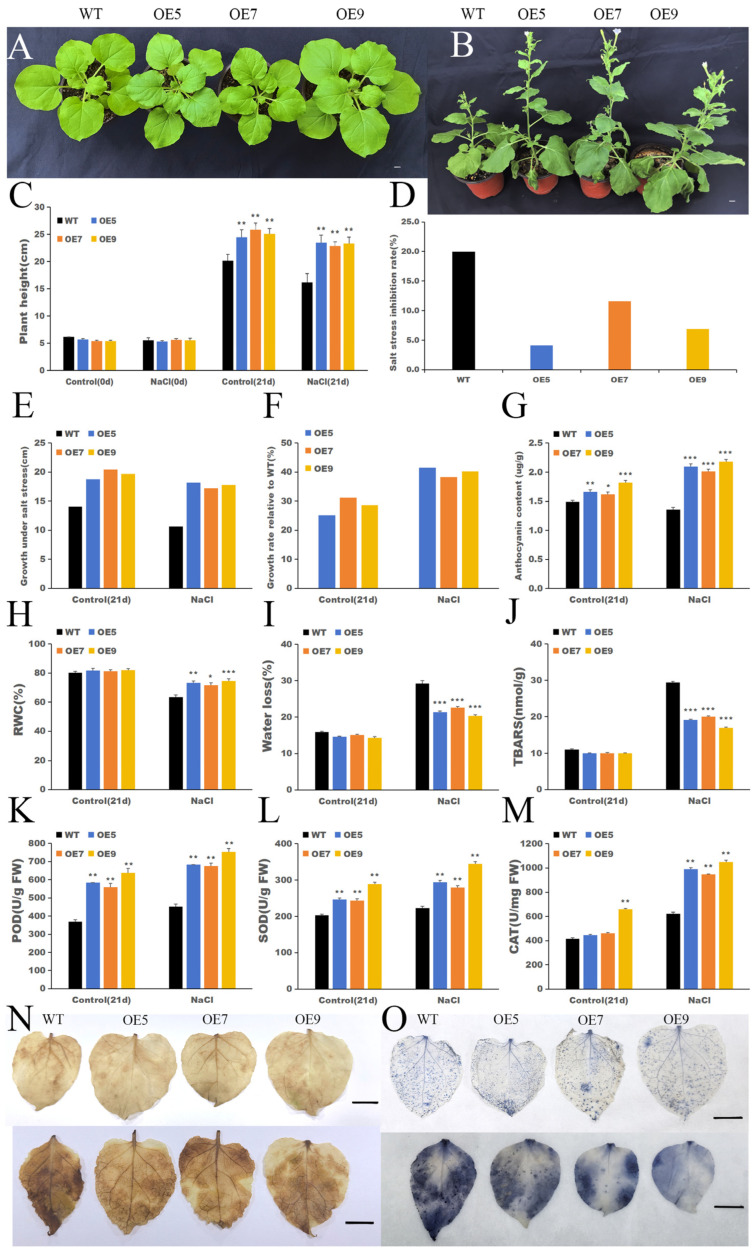
Physiological and biochemical analysis of WT and *IbHSF22* overexpressing plants exposed to control and salt treatment. (**A**,**B**) Plant phenotypes before and after stress treatment. The scale bar is 1 cm. Determination and analysis of: (**C**) Plant height; (**D**) Salt stress inhibition rate; (**E**) Growth under salt stress (cm); (**F**) Growth rate relative to WT; (**G**) Anthocyanin content (μg/g); (**H**) RWC; (**I**) Excised leaf water loss; (**J**) TBARS content; (**K**) POD activity; (**L**) SOD activity; (**M**) CAT activity; (**N**) DAB staining; (**O**) NBT staining. The scale bar is 1 cm, * *p* < 0.05, ** *p* < 0.01, *** *p* < 0.001 versus the WT under identical conditions (Student’s *t*-test; mean ± SD, n = 3).

Before salt stress treatment, compared with the WT, *IbHSF22* transgenic plants showed more obvious leaf pigment accumulation at multiple developmental stages. To evaluate the function of *IbHSF22* in salt stress response, three transgenic lines (*IbHSF22*-OE5, *IbHSF22*-OE7, and *IbHSF22*-OE9) were subjected to salt stress together with WT plants. Salt stress caused typical salt damage symptoms in WT plants, including restricted expansion of new leaves, significant wrinkling and wilting of mature leaves, and overall growth was significantly inhibited; however, the transgenic lines showed stronger growth vigor and less phenotypic changes (Figure 9A). Specifically, after 21 days of stress treatment, the plant height of all plants decreased, but the inhibition rate of the transgenic lines (4.08–6.91%) was significantly lower than that of the WT (19.97%). Under control conditions, the growth increment in the transgenic lines was 18.80 to 20.43 cm, and under salt stress conditions, it was 17.23 to 18.17 cm, while the growth increments in the WT under the two conditions were 14.07 cm and 10.63 cm, respectively. Compared with the WT, the growth rate of the transgenic plants under normal conditions increased by 25.18% to 31.16%, and under salt stress conditions, it increased by 38.30% to 41.48%. These results indicate that *IbHSF22*-OE can effectively promote vegetative growth under salt stress and significantly enhance the stress tolerance of plants.

Quantitative analysis showed that the anthocyanin content in the three transgenic lines was significantly higher than that in the WT under salt stress conditions. Notably, the anthocyanin content in the WT was slightly lower after stress than in the non-stressed control (Figure 9G). To further elucidate the physiological mechanisms of their improved salt tolerance, we conducted a systematic examination of multiple biochemical and physiological indicators. The results showed that, in addition to a higher plant height, the RWC under salt stress was significantly increased (Figure 9H). Moreover, the activities of the antioxidant enzymes CAT, POD, and SOD were significantly enhanced under salt stress (Figure 9K–M). In contrast, the transgenic lines had a significantly reduced water loss rate and a lower TBARS content (Figure 9I,J), indicating a lower degree of membrane lipid peroxidation and better maintenance of cell membrane integrity. In addition, histochemical localization analysis of ROS using DAB and NBT staining revealed that the accumulation of H_2_O_2_ and O_2_^−^ in the leaves of the WT was significantly higher than in the transgenic plants (Figure 9N,O), indicating that transgenic lines have a stronger capacity for scavenging ROS. In summary, *IbHSF22*-OE significantly improved the salt tolerance of the transgenic plants, and the mechanism may involve improvement in osmotic adjustment ability, maintenance of cell membrane structure, and coordinated enhancement in the ROS scavenging system.

### 2.9. IbHSF22 Modulates the Transcription of Genes Involved in Anthocyanin Biosynthesis Under Salt Stress

**Figure 10:**
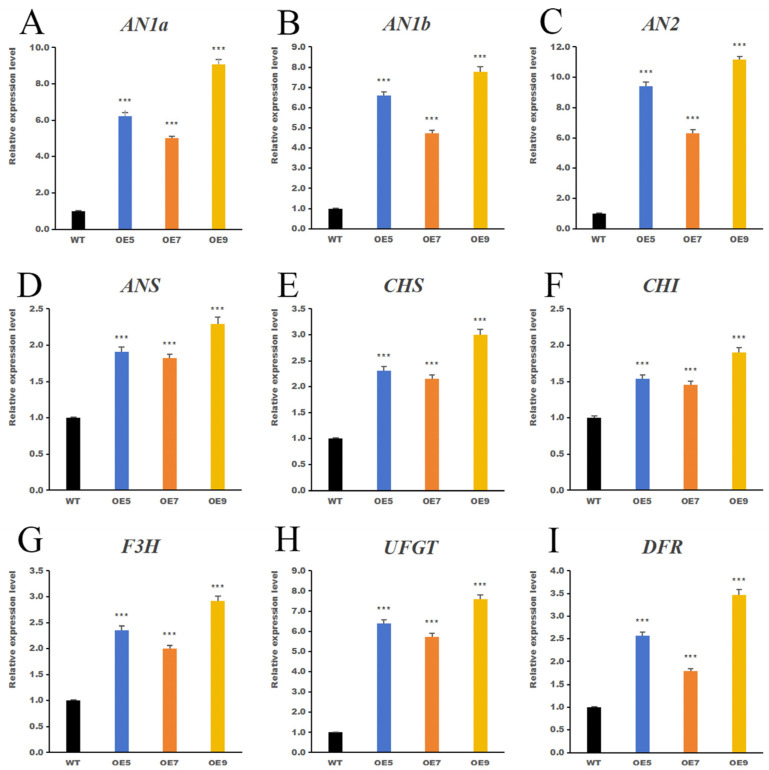
Under control conditions (21 d), the expression of key anthocyanin biosynthesis genes was compared between *IbHSF22*-OE lines and the WT. Subfigures show the relative expression levels of: (**A**) *AN1a*, (**B**) *AN1b*, (**C**) *AN2*, (**D**) *ANS*, (**E**) *CHS*, (**F**) *CHI*, (**G**) *F3H*, (**H**) *UFGT*, and (**I**) *DFR*. Values are mean ± SD (n = 3; *** *p* < 0.001 vs. WT, Student’s *t*-test).

**Figure 11:**
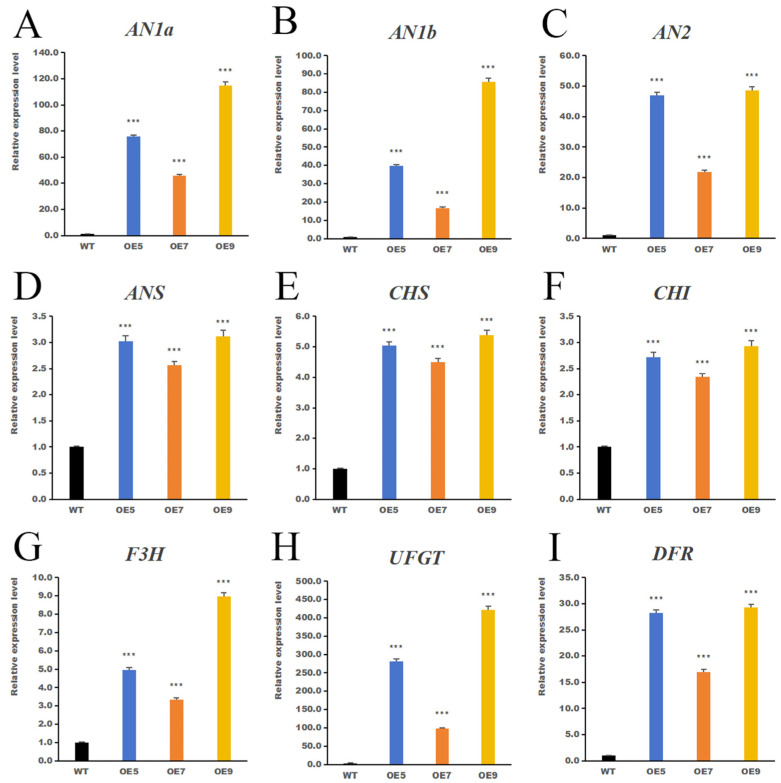
Under salt stress, the expression of key anthocyanin biosynthesis genes was compared between *IbHSF22*-OE lines and the WT. Subfigures show the relative expression levels of: (**A**) *AN1a*, (**B**) *AN1b*, (**C**) *AN2*, (**D**) *ANS*, (**E**) *CHS*, (**F**) *CHI*, (**G**) *F3H*, (**H**) *UFGT*, and (**I**) *DFR*. Values are mean ± SD (n = 3; *** *p* < 0.001 vs. WT, Student’s *t*-test).

To further elucidate the molecular mechanism by which *IbHSF22* enhances anthocyanin biosynthesis, we analyzed the transcript levels of key structural genes and transcription factors in the anthocyanin pathway in WT and *IbHSF22*-OE lines under control and salt-stress conditions (Figure 10 and Figure 11).

Under normal conditions, the expression of most of the anthocyanin-related genes was significantly increased in the *IbHSF22*-OE lines compared to the WT under control (21 d) conditions. In particular, the regulatory genes *AN1a*, *AN1b*, and *AN2*, and the late biosynthetic gene *UFGT* were highly upregulated, with transcript levels of 5 to 11 fold higher in the OE lines.

Salt stress promoted strong transcriptional activation of the anthocyanin pathway in *IbHSF22*-OE plants. The most dramatic induction occurred in *UFGT*, whose expression levels were elevated to about 98-fold (OE7), 281-fold (OE5), and 422-fold (OE9) compared to those in the stressed WT. The Transcription factors *AN1a* and *AN1b* exhibited high induction, with the transcript levels observed to be 115-fold and 86-fold higher, respectively. Other key structural genes, such as *DFR*, *F3H*, *CHS*, *CHI*, and *ANS*, were also consistently and significantly upregulated in all three transgenic lines under salt stress.

Thus, these results show that *IbHSF22* is a strong transcriptional activator of the anthocyanin biosynthetic pathway, which acts to constitutively enhance the expression of pathway genes while also massively amplifying their salt-induced expression, most notably for the last and crucial step catalyzed by the UFGT enzyme.

### 2.10. IbHSF22 Modulates the Transcription of Genes Involved in Stress Responses

**Figure 12:**
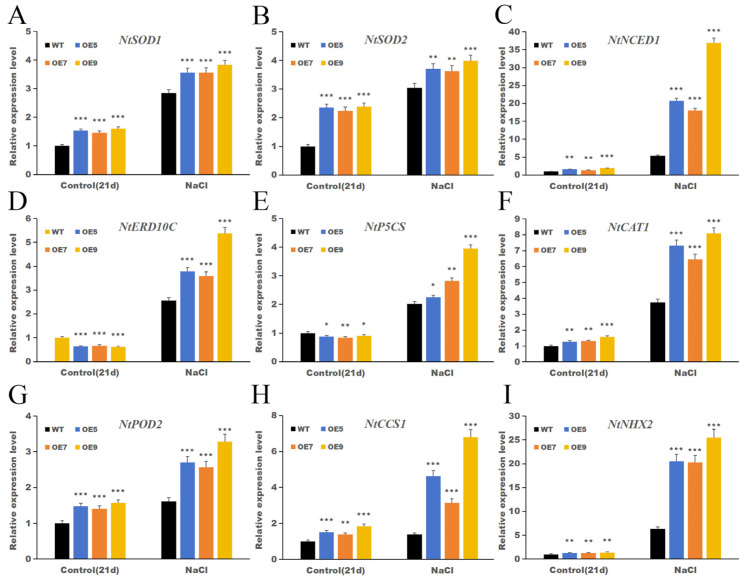
Under salt stress, the expression patterns of genes involved in the antioxidant and stress-responsive genes in transgenic plants overexpressing *IbHSF22* and WT plants. Subfigures show the relative expression levels of: (**A**) *NtSOD1*, (**B**) *NtSOD2*, (**C**) *NtNCED1*, (**D**) *NtERD10C*, (**E**) *NtP5CS*, (**F**) *NtCAT1*, (**G**) *NtPOD2*, (**H**) *NtCCS1*, and (**I**) *NtNHX2*. Values are mean ± SD (n = 3; * *p* < 0.05, ** *p* < 0.01, *** *p* < 0.001 versus the WT under identical conditions, Student’s *t*-test).

In order to check whether *IbHSF22* modulates the transcriptional programs other than anthocyanin biosynthesis, mediated by salt stress, expression profiles of some key stress-responsive genes were analyzed in WT and *IbHSF22*-OE lines under control and salt-stress conditions (Figure 12).

In the *IbHSF22*-OE lines, the transcript levels of most of the stress-related genes under normal growth conditions were comparable to those in the WT, and only very minor increases were detected.

The expression of all stress-responsive genes investigated in this study was induced by salt stress in both WT and transgenic plants, but the extent of induction was much greater in the *IbHSF22*-OE lines. Accordingly, the most strongly induced genes were *ERD10C* and *NHX2*. Thus, *ERD10C*, encoding a dehydrin protein involved in the protection of cellular functions from abiotic stress, showed a 20 to 37 fold increase in transcript level in the OE lines compared with the stressed WT. Similarly, *NHX2*, a vacuolar Na^+^/H^+^ antiporter playing a critical role in ion homeostasis, was induced 20 to 26 fold in the transgenic lines. The transcript abundance of genes coding for the core antioxidant enzymes *SOD1*, *SOD2*, *CAT1*, and *POD2* was also consistently and significantly higher in the OE lines than in WT under salt stress. Moreover, both *P5CS*, a key gene involved in proline biosynthesis, and *NCED1*, a rate-limiting gene in ABA biosynthesis, were substantially more highly expressed in the transgenic plants than in WT when the plants were subjected to salt stress.

Collectively, these findings point to *IbHSF22* being a broad-spectrum transcriptional regulator that coordinately modulates multiple components of the salt stress response, including osmotic adjustment, oxidative stress detoxification, and ion homeostasis.

## 3. Discussion

### 3.1. Identification and Evolutionary Analysis of the IbHSFs

Heat shock factors (HSFs) are structurally and functionally conserved master regulators of plant abiotic stress responses [10,14]. In this study, we identified 40 *IbHSF* genes from the sweet potato genome. Phylogenetic analysis classified them into three subfamilies (A, B, and C), with the majority belonging to subfamily A and only *IbHSF9* assigned to subfamily C. This distribution pattern aligns with observations in *Populus trichocarpa* [10], *Actinidia chinensis* [14], *Salvia miltiorrhiza* [18], *Asparagus officinalis* [19], and *Astragalus mongholicus* [20], underscoring the high evolutionary conservation of the *HSF* family in higher plants.

Gene structure and conserved motifs analyses showed that members within the same subfamily share highly similar exon-intron organizations and motif compositions, implying functional redundancy or cooperative functions [13]. All IbHSF proteins possess a typical DNA-binding domain (DBD) consisting of Motifs 1–3, which is an evolutionarily conserved characteristic of HSFs across plant lineages [21]. To support functional coherence among phylogenetically related HSFs, Yang et al. [13] demonstrated that HSFs within the same subfamily in *Mangifera indica* exhibit relatively similar motif architectures and participate in the regulation of phenolic compound biosynthesis. In addition, synteny analysis identified 17 pairs of segmentally duplicated *IbHSF* genes, implying that segmental duplication has played a major driving role in the expansion of this gene family. This mechanism is prevalent in plants, as evidenced by studies in *Phoebe zhennan* [12], *Secale cereale* [22], and *Citrus sinensis* [23], which collectively highlight the central role of segmental duplication in *HSF* family evolution.

### 3.2. IbHSF22 Positively Regulates Anthocyanin Biosynthesis and Accumulation

HSFs are major regulatory factors controlling the complex network of anthocyanin biosynthesis in various plant species [24]. Anthocyanins, a major subclass of flavonoids, are synthesized under finely tuned transcriptional control mediated by HSFs, both directly and indirectly. In several species, evidence exists for the involvement of HSFs in activating the anthocyanin pathway. For example, in chrysanthemum, under heat stress, there is a close match between the expression of the *HSF* gene *Cse_sc000572.1_g090.1* and the accumulation of anthocyanin [17]. In mango, HSFs interact with MYB and C2H2-type zinc finger proteins to enhance light-induced anthocyanin production [25], while in strawberry, certain HSFs take part in pigment metabolism through the ABA signaling pathway [26]. In the *European pear*, HSFs have also been identified as candidate transcriptional regulators of anthocyanin synthesis [27]. An important functional proof was given by Park et al. [28]. Their work showed that direct induction of *HSF* expression via a CRISPR/dCas9 system in *Arabidopsis* plants led to massive anthocyanin accumulation, indicating a direct role of HSFs in the regulation of flavonoid metabolism. Moreover, HSFs can influence not only anthocyanin-specific pathways but also the broader flavonoid network, thereby indirectly affecting anthocyanin biosynthesis. In soybean, *GmHSFB2b* upregulates the flavonoid pathway and, consequently, enhances tolerance to abiotic stress [15]. In kiwifruit, several members of the B-subfamily HSFs (*AeHSFB1c/1d/2c/3b*) are induced under stress and thus take part in flavonoid biosynthesis [14]. In poplar, *HSFA5a* binds directly to promoters of flavonol-related genes such as *FLS1/4*, *CHS1*, and *F3′H2* to catalyze flavonol biosynthesis while enhancing oxidative stress tolerance [16]. Collectively, these findings place HSFs at the center of the transcriptional network regulating anthocyanin biosynthesis and thus provide a solid conceptual framework for investigating *IbHSF22*-mediated anthocyanin accumulation in sweet potato.

As indicated in Results, *IbHSF22* was selected for in-depth study due to its strong differential expression correlated with anthocyanin accumulation. Phylogenetically, it belongs to the *HSF* B2 subgroup and carries the conserved structural features of *HSF* genes: a DBD responsible for the recognition of the HSE, an oligomerization domain HR-A/B, which is essential for trimerization, an NLS, and a C-terminal AHA motif. While the AHA motif is commonly lacking in the members of subgroup B2 [29], its presence in *IbHSF22* was confirmed by bioinformatics analysis. The B2 subgroup acts in cooperation with A-class HSFs to control the expression of heat shock protein genes functionally, maintaining a balance between stress responses and normal growth/development in plants [2]. To study the biological function of *IbHSF22*, we performed subcellular localization and functional assays. Subcellular localization confirmed this gene’s nuclear localization as predicted for a transcription factor [19]. Functional studies in transgenic *Nicotiana benthamiana* showed that *IbHSF22*-OE significantly increased anthocyanin accumulation in these plants from two to three times that of WT plants (*p* < 0.01). In addition, the major structural genes in the anthocyanin biosynthetic pathway, including *NtCHS*, *NtF3H*, *NtDFR*, and *NtANS*, have been strongly increased within the overexpression lines. These results strongly indicate that *IbHSF22* acts as a positive transcriptional regulator, leading to enhanced anthocyanin production through direct or indirect activation of the expression of important biosynthetic genes.

Contrary to the general repressive role associated with the B2 subgroup, our findings identify *IbHSF22* as a positive regulator in anthocyanin biosynthesis. This atypical function may be attributed to its distinct structural characteristics and context-dependent regulation. A key structural feature is its C-terminal AHA motif, which is seldom found in conventional B2 subgroup proteins [29] and likely provides a molecular basis for transcriptional activation. Additionally, the regulatory output of HSFs is not fixed but can be reshaped by cellular context, protein–protein interactions, or post-translational modifications, enabling a shift from repression to activation. This view is supported by prior studies showing that certain B-class HSFs, such as *GmHSFB2b* [15] and *AeHSFB* [14], can positively regulate stress-responsive flavonoid metabolism, underscoring the functional plasticity within this subclass.

### 3.3. IbHSF22 Enhances Salt Tolerance in Nicotiana benthamiana Through Coordinated Activation of Multiple Pathways

The HSFB subfamily, especially its B2 subgroup, has a key role in the response of plants to salt and drought stresses, even as functional divergence between species takes place. More and more studies have identified positive regulatory members of this subgroup that contribute to the abiotic stress tolerance of plants via different molecular mechanisms. *GmHSFB2b* from soybean enhances salt tolerance by inducing the flavonoid biosynthesis pathway and reducing the accumulation of ROS [15]. *CaHSFB2* from chickpea improves the resistance to drought through the enhanced expression of key stress-responsive genes, including *RD22*, *RD26*, and *RD29A* [30]. *TaHSFB4-2B* or *TaHsfB2d* overexpression enhances tolerance to salinity and drought in transgenic Arabidopsis [30,31]. Meanwhile, *VpHSF1* from Chinese wild grape (*Vitis pseudoreticulata*) enhances thermotolerance and drought resistance in tobacco [32]. In addition, in kiwifruit, class A HSF *AeHSFA2b*, synergistic in function with B-type HSFs, increases salt tolerance by directly activating the expression of *AeRFS4* [14], and *HSFA5a* enhances the salt resistance of poplar through regulation of flavonol biosynthetic genes and suppression of ROS accumulation in lateral roots [16]. All these support the view that B2-type HSFs, along with their functionally associated homologues, reinforce plant stress resilience by simultaneously inducing antioxidant biosynthesis, modulating ABA signaling, and inducing osmotic adjustment pathways.

Building on this conserved mechanism and considering the established role of anthocyanins in the stress response, we identified a salt stress tolerance function for *IbHSF22* that had not been characterized previously. Plants of *IbHSF22*-OE *Nicotiana benthamiana* lines displayed higher growth vigor and greater relative water content, as well as higher soluble protein content under saline conditions. The critical activities of antioxidant enzymes, such as SOD, POD, and CAT, were enhanced, while the accumulation of TBARS and ROS was reduced in these plants, showing their enhanced capacity to mitigate oxidative injury and maintain membrane integrity. It should be noted that the TBARS assay, while a widely used indicator of lipid peroxidation, may also react with other aldehydes present in plant tissues [33].

At the molecular level, overexpression of *IbHSF22* triggered a broad transcriptional network related to stress adaptation, including genes encoding antioxidant defense genes (*SOD1*, *CAT1*, *POD2*), osmotic adjustment machinery (*P5CS*), ABA biosynthesis machinery (*NCED1*), and ion homeostasis machinery (*NHX2*). Of particular interest was that *IbHSF22* also induced a set of core regulatory genes in the anthocyanin pathway, including *AN1a*, *AN1b*, and *AN2*, as well as downstream structural genes. Since anthocyanins function as powerful antioxidants [5,34,35,36,37], we thereby suggest a synergistic model: *IbHSF22* coordinates both direct stress defense mechanisms and metabolic pathways contributing to redox balance. In this model, anthocyanin accumulation was not only a secondary metabolic outcome but also part of the stress response, amplifying the overall oxidative stress tolerance of the plant. This dual-function mechanism is supported by multiple studies: anthocyanin levels show a positive correlation with salt tolerance in *Arabidopsis thaliana* and *Brassica napus* [5,38,39] highlighted the role of anthocyanins as ROS scavengers under abiotic stress; Chutipaijit et al. [37] found that anthocyanin and proline co-accumulated in the salt-tolerant genotypes of *Oryza sativa*, leading to cellular stability; *MYB112* was shown to promote anthocyanin biosynthesis under the action of both salinity and high light in *Arabidopsis thaliana* [35]; and ABA-induced anthocyanin accumulation via H_2_O_2_ signaling was linked with enhanced resilience to stress [34]. Moreover, in general, salt-tolerant varieties of *Oryza sativa* are characterized by the enhanced level of anthocyanin and proline accumulation [37].

## 4. Materials and Methods

### 4.1. Plant Material and Growth Conditions

Tobacco (*Nicotiana benthamiana*) seedlings were cultivated in a plant growth chamber under a 16 h/8 h light/dark cycle at 25 °C and 60% relative humidity. The growth medium was a sterilized mixture of peat soil, vermiculite, and perlite in a 1:1:1 (*v*/*v*/*v*) ratio. The plant expression vector pCAMBIA1304 and *Agrobacterium tumefaciens* strain GV3101 were maintained in our laboratory.

### 4.2. Identification and Characterization of the HSF Gene Family in Sweet Potato

The genome sequence and corresponding annotation files of sweet potato (*Ipomoea batatas* L.) were obtained from the Ipomoea Genome Hub database (https://sweetpotao.com/; accessed on 2 December 2023). The method used in discovering the sweet potato was carried out by the HMM search of the PFAM HSF domain (PF00447) held in the Pfam database (http://pfam.xfam.org/; accessed on 2 July 2024). The candidates’ genes were screened by TBtools-II v2.136 [40]. The putative *IbHSF* genes were further verified by BLASTp analysis against the NCBI database (https://www.ncbi.nlm.nih.gov/; accessed on 2 July 2024) and the PlantTFDB (https://planttfdb.gao-lab.org/; accessed on 2 July 2024). Confirmation of protein domains was performed using NCBI-CDD (https://www.ncbi.nlm.nih.gov/Structure/bwrpsb/bwrpsb.cgi; accessed on 2 July 2024) and Pfam (https://pfam.xfam.org/; accessed on 2 July 2024). A total of 40 genes were identified and designated as *IbHSF1* to *IbHSF40*; the original gene names and their corresponding new names of sweet potato and *Arabidopsis thaliana* are given in Table S2.

### 4.3. Evolutionary Analysis and Gene Structure of IbHSFs

The conserved motifs in IbHSF proteins were identified by using the MEME web tool (https://meme-suite.org/meme/tools/meme; accessed on 3 July 2024) and setting the number of motifs to a maximum of 10. Conserved domains were predicted by using the NCBI Batch Web CD-Search tool (https://www.ncbi.nlm.nih.gov/Structure/bwrpsb/bwrpsb.cgi; accessed on 3 July 2024). The gene structure graphic was obtained by using the “Visualize Gene Structure” module in TBtools-II v2.136 software with the GFF3 file containing the sweet potato genome annotation, through which the gene structure graphic was generated. The standard presentation includes coding regions (CDS) that are depicted by a yellow box, an untranslated region (UTR) depicted by a green box, and an intron depicted by lines [40].

### 4.4. Physicochemical Characteristics and Subcellular Localization

The theoretical physicochemical parameters of IbHSF proteins, including molecular weight, theoretical isoelectric point (pI), and grand average of hydropathicity (GRAVY), were calculated using Protein Parameter Calc in TBtools-II v2.136 [40]. The subcellular localization was predicted using WoLF PSORT. https://wolfpsort.hgc.jp/. Accessed on 18 December 2024. For experimental verification, the coding sequence of *IbHSF22* was fused with the pBinGFP4 vector to generate the recombinant plasmid *IbHSF22-pBinGFP4*, which was then transformed into *Agrobacterium tumefaciens* GV3101. Transient expression in tobacco epidermal cells was performed by co-infiltration with the nuclear marker *H2B-RFP*. After incubation for 24 h in the dark at 25 °C, plants were transferred to normal light/dark conditions. Observation of leaf samples under a confocal microscope (Leica Microsystems, Wetzlar, Germany) was made at 72 h post-infiltration.

### 4.5. Phylogenetic Analysis

HSF protein sequences from sweet potato and *Arabidopsis thaliana* were obtained from PlantTFDB (https://planttfdb.gao-lab.org/; accessed on 3 July 2024). For multiple sequence alignment, ClustalW was used; the phylogenetic tree was then constructed using MEGA11 (V11.0.13) with the Maximum Likelihood approach following the JTT model, and a bootstrap analysis with 1000 replicates was performed. The tree was visualized and annotated using iTOL (https://itol.embl.de/; accessed on 10 July 2024).

### 4.6. Collinearity and Duplication Analysis

The genome sequences and corresponding annotation data for *Ipomoea trifida* (ASM357666v1), *Ipomoea triloba* (ASM357664v1.59), *Arabidopsis thaliana* (TAIR10.59), *Solanum lycopersicum* (SL3.0.59), *Brassica rapa* (Brap_v3.01), *Brassica oleracea* (BOL.59), *Capsicum annuum* (ASM51225v2.59), and *Oryza sativa* (ASM143393v1) were downloaded from the corresponding authoritative databases, including NCBI GenBank/Assembly, TAIR, Ensembl Plants, Sol Genomics Network (SGN), and BRAD. Collinearity analysis between the 40 *IbHSFs* and their orthologous counterparts in the selected plant species (see gene accession numbers in Table S4) was performed using the MCScanX tool in TBtools-II v2.136 [40]. It identified segmental and tandem duplication events, and the results were visualized using the Advanced Circos function of TBtools.

### 4.7. The Acquisition and Screening of Transgenic Tobacco

Transgenic tobacco plants overexpressing *IbHSF22* were produced by *Agrobacterium tumefaciens*-mediated genetic transformation using the GV3101 strain. The detailed experimental procedures were performed as described by Kim et al. [41]. Genomic DNA was extracted from putative transgenic lines. Positive transformants were initially screened by PCR using gene-specific primers. Thereafter, the transcriptional levels of *IbHSF22* in the PCR-positive plants were quantified by qRT-PCR. *NtActin* was used as the internal control gene for data normalization. The relative gene expression levels were calculated based on the 2^−ΔΔCt^ method [42]. Three independent T_0_ transgenic lines showing the highest expression levels of *IbHSF22* were selected. Homozygous T_2_ progeny was obtained through successive self-pollination.

### 4.8. Stress Treatment of Transgenic Tobacco

For the stress assays, four-week-old wild-type (WT) and homozygous T_2_ plants were exposed to 21 days of salt stress by irrigation with 100 mM NaCl [43]. Two control groups were used in order to accurately dissect the effects of salt stress: the initial control (Control (0 d)) was harvested at the time of the start of the stress treatment in order to provide a baseline, and the concurrent control (Control (21 d)) was kept under normal conditions for the duration of the experiment in order to take into account any plant developmental effects. Each experimental group included six biological replicates, and the entire experiment was independently repeated three times. Throughout the period of stress, phenotypic responses were observed and recorded.

### 4.9. Physiological Analysis

The level of lipid peroxidation was estimated by measuring thiobarbituric acid reactive substances (TBARS) using the TBA assay, following the method described by [33], with the understanding that this method detects TBARS as a broad indicator, not MDA exclusively. SOD activity was assayed by determining its capacity to inhibit photochemical reduction of nitroblue tetrazolium (NBT) according to [44]. Peroxidase (POD) activity was detected according to the oxidation of guaiacol method [45]. Catalase (CAT) activity was measured by using a commercial kit (Solarbio, Beijing, China). Hydrogen peroxide and superoxide anion were also localized histochemically with 3,3′-diaminobenzidine (DAB) and nitroblue tetrazolium (NBT), respectively, according to [45].

### 4.10. Determination of Anthocyanin Content

Anthocyanin content was quantified based on the method described by Yang et al. [46] with some modifications. Generally, frozen leaf samples were ground in liquid nitrogen and extracted with 1 mL of 1% (*v*/*v*) HCl-methanol at 4 °C in darkness for 24 h. Then, the absorbance of the supernatant after centrifugation was measured at 530 nm and 657 nm. Anthocyanin content was calculated according to the formula [47]:

Anthocyanin content = (A_530_ − 0.25 × A_657_)/fresh weight.

### 4.11. RNA Extraction and Real-Time Quantitative PCR (RT-qPCR) Analysis

Total RNA was extracted from three biological replicates using a Plant RNA Extraction Kit (R1200, Solarbio, Beijing, China). The RNA concentration and purity were checked on a NanoDrop spectrophotometer. Acceptable A_260_/A_280_ ratios were considered to be between 1.8 and 2.1, and A_260_/A_230_ values should be ≥2.0. The integrity of the RNA was checked by electrophoresis on a 1% agarose gel. Contaminating genomic DNA was removed by DNase treatment, and first-strand cDNA was synthesized from 1 μg total RNA using the PrimeScript RT Reagent Kit (RR047A, TaKaRa, Beijing, China). RT-qPCR was carried out in TB Green Premix Ex Taq II (RR820A, TaKaRa) using a QuantStudio 6 Flex Real-Time PCR System (Applied Biosystems, Waltham, MA, USA). *NtActin* was used as the internal control, and relative transcript levels were determined using the 2^−ΔΔCt^ method [42]. All reactions were performed in triplicate (three technical replicates). Primer sequences are provided in Supplementary Table S3.

## 5. Conclusions

A genome-wide investigation of the *HSF* gene family in sweet potato has revealed 40 *IbHSF* genes that have evolved mainly through segmental duplication. Functionality analysis has demonstrated that *IbHSF22* is a transcription activator, simultaneously promoting anthocyanin production and enhancing resistance to salt stress. It achieves this by activating the primary genes responsible for anthocyanin production and a unified system of antioxidant responses. Therefore, *IbHSF22* brings together the initial stress resistance mechanisms and secondary metabolic pathways. *IbHSF22* is a vital gene target that should be utilized in improving the adaptability of sweet potato.

## Data Availability

Data are contained within this article and Supplementary Materials.

## Supplementary Materials

The following supporting information can be downloaded at: https://www.mdpi.com/article/10.3390/plants15020236/s1, Table S1: Basic information of *HSF* family genes in sweet potato; Table S2: The list of gene renamings in this study; Table S3: The primers used in this study; Table S4: Orthologous *HSF* genes between sweet potato and other plants.
